# Sustainability of health services in refugee hosting districts: a qualitative study of health services in three west Nile refugee hosting districts, Uganda

**DOI:** 10.1186/s13031-023-00507-y

**Published:** 2023-03-10

**Authors:** Henry Komakech, Christopher Garimoi Orach, Lynn M. Atuyambe

**Affiliations:** Department of Community Health, and Behavioural Science, Makerere University School of Public Health, P. O. Box 7072, Kampala, Uganda

**Keywords:** Sustainability, Health services, Humanitarian health assistance, West Nile, Uganda

## Abstract

**Background:**

Humanitarian health assistance programmes have expanded from temporary approaches addressing short-term needs to providing long-term interventions in emergency settings. Measuring sustainability of humanitarian health services is important towards improving the quality of health services in refugee settings.

**Objective:**

To explore the sustainability of health services following the repatriation of refugees from the west Nile districts of Arua, Adjumani and Moyo.

**Methods:**

This was a qualitative comparative case study conducted in three west Nile refugee-hosting districts of Arua, Adjumani, and Moyo. In-depth interviews were conducted with 28 purposefully selected respondents in each of the three districts. Respondents included health workers and managers, district civic leaders, planners, chief administrative officers, district health officers, project staff of aid agencies, refugee health focal persons and community development officers.

**Results:**

The study shows that in terms of organization capacity, the District Health Teams provided health services to both refugee and host communities with minimal support from aid agencies. Health services were available in most former refugee hosting areas in Adjumani, Arua and Moyo districts. However, there were several disruptions notably reduction and inadequate services due to shortage of drugs and essential supplies, lack of health workers, and closure or relocation of health facilities in around former settlements. To minimize disruptions the district health office reorganized health services. In restructuring health services, the district local governments closed or upgraded health facilities to address reduced capacity and catchment population. Health workers employed by aid agencies were recruited into government services while others who were deemed excess or unqualified were laid off. Equipment and machinery including machines and vehicles were transferred to the district health office in specific health facilities. Funding for health services was mainly provided by the Government of Uganda through the Primary Health Care Grant. Aid agencies, however, continued to provide minimal support health services for refugees who remained in Adjumani district.

**Conclusion:**

Our study showed that while humanitarian health services are not designed for sustainability, several interventions continued at the end of the refugee emergency in the three districts. The embeddedness of the refugee health services in the district health systems ensured health services continued through public service delivery structures. It is important to strengthen the capacity of the local service delivery structures and ensure health assistance programmes are integrated into local health systems to promote sustainability.

## Introduction

According to the United Nations High Commission for Refugees (UNHCR), an estimated 79.5 million people were displaced worldwide at the end of 2020. This included 26 million refugees and 45.7 million Internally Displaced Persons (UNHCR 2020). In recent years, there has been an increase in the number of refugees living in protracted situations. By the end of 2019, an estimated 15.7 million 77% of the refugee population were in exile for more than five years. According to the United Nations High Commission for Refugees (UNHCR), 35 countries most affected by protracted refugee displacements are low-income countries. Concerns over protracted refugee situations have become prominent for several reasons. Firstly, host governments and aid agencies in developing countries often struggle to meet the protection and assistance needs of refugees. Additionally, there has been a significant decline in funding for humanitarian assistance due to the reduction in international aid and challenging multilateral cooperation [15]. These, and a combination of other factors have affected the provision of protection and assistance in protracted refugee situations.

Uganda has been hosting refugees for several decades. The first influx of 100,000 refugees in West Nile region happened between 1983–1994 into Arua district. Between 1986–1994, an estimated 210,000 Sudanese refugees were settled in the districts of Arua, Moyo and Adjumani in the west Nile region [13]. Most of the refugees lived in the west Nile region for over two decades where protection and assistance services were provided by aid agencies and the government. The health systems in refugee hosting districts have always had inadequate capacity to meet the health needs of the refugees and nationals. In a bid to address the health needs of refugees, the UNHCR in collaboration with international non-government organization (NGO) established health service delivery structures in the districts of Adjumani, Arua and Moyo. Through this arrangement, health services were provided by local and international NGOs with funding through the UNHCR [22]. Humanitarian health assistance in Uganda have been characterized by vertical programs.

Protracted refugee situations raise important questions about the sustainability of humanitarian assistance programmes in developing countries. It has become an important theme for governments, international NGOs, donors, fundraisers, beneficiaries, and other stakeholders. Humanitarian assistance programs are implemented on the assumption that the emergencies will come to an end while ensuring that the protection and assistance needs of the affected population are met. However, humanitarian assistance programmes are associated with short-term interventions mainly focussing on saving lives and alleviating suffering in situations of extreme stress and crises when local actors have inadequate capacities. Discussions surrounding sustainability have centred around inadequate funding, end of an emergency, and whether or not the problems the interventions were designed for have been addressed [33]. However, little attention has be paid to the sustainability of health services in in refugee settings in low-income countries. 

Few studies have examined the sustainability of health services in refugee settings. A study by [32] found that sustainability was enhanced through the development of operations, maintenance, and administrative strategy to reflect and maximise the health interventions in Jordan. While in Afghanistan, the Ministry of Public Health provided emergency health services while rebuilding the health system [18]. An evaluation of the UNHCR’s Quick Impact Projects in Mozambique showed that aid agencies encouraged community participation that ensured smooth transition of interventions to local authorities. Several agencies stayed for over 15 years, shifting from humanitarian aid to development-oriented activities [20]. Overall, lack of evidence in this field may be attributed to two factors, conceptual and methodological and practical challenges. Methodologically, there are differing opinions about sustainability from various disciplines, time frames and context [27]. From a practical perspective, aid agencies are caught between two diverging roles, providing protection and assistance often as short to medium term objectives, and undertaking developmental initiatives.

Given the limited information, an assessment of the sustainability of health services in refugee hosting areas is important to inform planning, design and implementation aimed at improving the quality of health service. This study explored the sustainability of health services in the west Nile refugee hosting districts in Uganda. This is important to inform policy makers and program managers on strategies to improve the provision of effective health services in refugee hosting areas.

### Framework for assessing sustainability

Sustainability has been used to mean different things by practitioners and disciplines. Available definitions highlight the following characteristics: the persistence of the innovation itself; and/or innovation-related benefits; and/or the development of the innovation or the context over time [17, 25]. A systematic review of sustainability literature from Low-Income Countries reported several definitions including both programmatic and financial components of sustainability [12]. These studies provide different conceptions and descriptions of sustainability divided into interventions focusing on maintenance: a) health benefits (utilitarian) and b) community capacity (empowerment). Several factors affecting sustainability have been identified including availability of resources (financial, human and equipment), perceptions of value of the program, collaboration and partnerships, leadership, community ownership, capacity of health systems, and ecological factors [5, 11, 26] [8].

Several frameworks have been advanced to describe and understand the sustainability of healthcare services. In this study, we have adopted the definition based on the Child Survival Sustainability Assessment (CSSA) [24]. Sustainability refers to ‘a contribution to the development of conditions, enabling individuals, communities and local organizations to express their potential, improve local functionality, develop mutual relationships of support and accountability and decrease dependency on insecure resources (financial, human, technical, informational), for local stakeholders to negotiate their respective roles in the pursuit of health and development, beyond a project intervention’. The framework is preferred because of the composition of health services and other elements which are important in understanding the sustainability of health assistance programmes. The CSSA framework consists of three dimensions and six components of sustainability (see Table 1). A fourth dimension, funding has been added to help in examining sustainability in this study.

**Table 1:** Framework for assessment of sustainability of health services in the west Nile districts

Dimension	Components	
Organisational capacity	1.1 Organisational capacity: the capacity that is needed within local organisations to maintain local services and activities	Organisational capacity in this study includes the district health service management and performance while trying to meet the health needs of the population
	1.2 Organisational viability: the capacity of an organisation for continuing effectiveness, in particular organisational dependency/interdependency and interconnectedness	Organisational viability refers to inter-linkages, support and connections which a district health service holds with the MoH, aid agencies and other districts to ensure it provides health services. It reflects the district health service organizational dependency, or interdependency, in a given region and district
Funding	2.1 Concerned with having sufficient funding to continue programme objectives. It follows the trajectory of funding pre-programme to programme funding and to post programme funding outcomes	The framework incorporated funding into the organisational dimension, specifically organisational viability. However, we consider that it warrants separate consideration, considering the centrality of financing to programme sustainability
Health and social services	3.1 Health and social services approach: availability, cost, accessibility and appropriateness of services	Health services are defined in this study in the broadest terms, as composed of all aspects of the district health services. The includes quality and coverage of services. Coverage is not reported in this study as it is not integrated into routine planning and M&E. The assessment of quality of health service was deemed beyond the scope of the study
Community, social and ecological	4.1 Community competence: overlapping elements that affect the community such as social cohesion and collective efficacy – ‘community competence	‘Community competence’ is defined as ‘the functions of district health services (leadership, management, communication, responsiveness to the community) and connectedness with community they serve which contribute to the overall health outcomes of the community
	4.2 Ecological, human, economic, political and policy environment: national and regional economic and political policies, and ecological conditions	In this study policy and local, regional and international political climates strongly influence the ability of the district health services and community capacity

## Methods

### Study design

We used a qualitative comparative case study design. The design was deemed appropriate for an in-depth inquiry into health services and the sustainability in refugee settings. The design is suitable to assess the ‘how’ or ‘why’ questions about phenomena’s and to reflect changes of events over time [6]. It supports the exploration of individuals, organisations and communities ranging from simple to complex interventions [34]. The design is suitable for exploring phenomena’s that have not been studied before [29]. Further, the design facilitates exploration of the localized nature of the context of health service delivery for refugees and host communities. Each of the districts was treated as a distinct case. Qualitative approaches are appropriate to use in exploratory studies such as this one especially when little is known about the topic being examined and testable hypothesis cannot be specified [7].

### Study setting

We conducted the study in three west Nile refugee hosting districts of Adjumani, Moyo and Arua. Data were collected between December – February 2017. The three districts are largely rural with a combined population of 2,390,899 people. The districts have experienced two major refugee influxes from South Sudan. The first influx occurring during 1983-1995 and most recently from 2014 to date. Health services for refugee and host communities in the three districts are provided by a mixture of public and Private-Not-For Profit providers. First, the public health system is managed and coordinated by the District Health Team through a network of tiered health facilities. Through the second, health services for refugees have been funded by the UNHCR and provided by a network of health facilities managed by implementing partners.

### Study population

We used a purposive sampling strategy to recruit a rich sample [21] of respondents from the three study districts. This strategy aimed at enhancing credibility [2]. We recruited 28 key-informants from a broad cross-section of backgrounds, knowledge, and skills regarding provision of health services for refugees and host population in each of the three districts. Interviews were conducted with two broad categories of respondents. These included administrators and managers (*n* = 11) Chief Administrative Officers, District Planners, District Health Officers, and Finance Officers/Managers. Technical staff (*n* = 17) included district engineers, community development officers, refugee health focal persons, health workers, facility managers and project staff of NGOs.

### Data collection procedure

We conducted key informant interviews with 28 respondents in the three refugee hosting districts. A semi-structured interview guide with open-ended questions and focused probes was used to guide the interviews. The guide consisted of several themes including: (a) elements of health services, (b) organisational factors on sustainability of health services (c) community and ecological factors and (d) funding. The questions emerged from literature review and the framework for sustainability of health services adopted to humanitarian health assistance. Interviews were conducted by trained research assistants. Interviews were conducted in English. Each interview lasted approximately between one to one and half hours. All interviews were audio-recorded with notes taken by interviewers.

### Data analysis

Data analysis was iterative and consisted of reading the transcripts, coding, comparing, and contrasting emerging themes, and devising more inclusive themes. Thematic analysis was conducted that produced a codebook to guide the coding process. Initially, a subset of the interview transcripts (*n* = 5) representative of various participants were selected for review by the researcher and research assistants. Key ideas and recurrent themes, which were deemed as typical of sustainability, were identified, and noted. The developed codebook defined each theme category and specified limitations on the text where each code applied. Coding, sorting, and code validation procedures followed as described by [23]. We used a systematic process to aid the identification of associations, patterns, and emergent themes within the data.

## Results

We conducted 28 in-depth interviews with 3 Chief Administrative Officers, 3, District Planners, 3 District Health Officers and 2 Finance Officers/Managers. Technical staff (*n* = 17) included 1 district engineer, 3 community development officers, 3 refugee health focal persons, 4 health workers and 5 facility managers. Sustainability is understood and described by various respondents from all the three study districts. The results are presented under four themes: 1) organisational, funding, health and social services and community and ecological in keeping with the objectives and framework adopted to guide the study.

### Organisational capacity

The health services established by humanitarian agencies continued to support service delivery in the three districts following the repatriation exercise. These interventions including infrastructure (health facilities), vehicles, medical equipment, and other resources were transferred to the district health office. Several capacity building activities including training and development of human resource were implemented by humanitarian agencies and the government to ensure that health service delivery continued in the three districts. These interventions were maintained by the district local governments and continued to support health service delivery.

*“As a district, we made sure that the assistance by NGOs were well-looked-after. The health centres, ambulances, devices, and other resources were maintained, though with challenges. The NGOs contributed to service delivery by constructing health facilities, ambulance, drugs and supplies and technical support by recruiting and training health workers to the district after the repatriation. As managers, we also learnt how to manage refugee and host health services in the district.” (District Health Officer).*

To mitigate any disruptions to health service delivery, respondents from all the three districts mentioned that the District Local Government recruited, and re-trained health workers previously employed by aid agencies. This was due to the shortages of health workers in refugee established health facilities as all had their contracts terminated by the departing aid agencies. These efforts were reinforced by other capacity building initiatives by the central government and aid agencies in the districts. These contributed to strengthening the capacity of the DHT to provide health services to refugees and host communities after the repatriation exercise.

*“Together with NGOs and the District Health Services, to ensure that we continued to provide health services and the support of the government, the district recruited and provided trainings, and mentorship programs to ensure that the health workers were in position to provide services. This was very helpful because as a district we were able to maintain acceptable levels of health service delivery” (Refugee Health Focal Person)*

According to district staff, the DLGs and the DHTs re-organised health services to ensure the continuation of service delivery in the three districts. The reorganization meant that health facilities and other support structures were upgraded, downgraded, shut down and relocated depending on several factors. The reorganization was based on consultations between the district local government and the Ministry of Health. In reorganizing health facilities, several considerations were made including the structural conditions and working capacity of the health facilities, the catchment population, and the resource available in the districts to support and maintain health facilities.

*“The District Local Government, the DHT, and the health sub-districts, restructured health facilities by either lowering, elevating, moving, and even closing some. We did this mainly based on judgement of the population and the physical state of the health facility (some were temporary structures). Other concerns included meeting the needs of the people, state and working condition of facilities. All these were done while following the mandate and responsibilities of the District local Government in consultation with the MoH.” (District planner)*

Despite the increased involvement in health service delivery overtime, the DHTs continued to depend on aid agencies for support. Adjumani district continued to host refugees and received additional support in several areas of health service delivery. Specific support was provided towards the recruitment and payment of salaries of health workers as an means of strengthening the capacity of the DHT to deliver health services. In Arua and Moyo, respondents mentioned that the departure of aid agencies led to the reduction in support from aid agencies. This affected the quality and coverage of health services in refugee established health services. The operations of the health facilities presented several challenges to the managers, administrators, and health workers due to the shortage of drugs/medicines, and few health workers.

## Organisational viability

### Effectiveness and sustainability of health services

Respondents described how the establishment of health service structures in the refugee settlements by aid agencies offered the DHT opportunities to expand service delivery in remote areas that were underserved in all the three districts. During the establishment of the settlements, health facilities were constructed to support service delivery for refugees. These facilities served both refugees and host communities. The DHT’s in all the three districts took over the management of the health facilities. These were used to increase and support health service delivery in underserved areas. Several health facilities were established in areas that otherwise did not have any services before in Adjumani district. While in Arua and Moyo districts, the DHT’s strengthened service delivery points in facilities that had been transferred to their authority.

*“Well, the refugee health service delivery structures were important in this district before and after repatriation and departure of NGO’s. During the establishment of the facilities, it provided much needed services in disadvantaged areas. We were able to increase service delivery in areas that were underserved in the district. Of course, we would not have been able to achieve these without the presence of refugees and NGO’s.” (District Health Officer)*

Overall, health service delivery improved because of interventions by aid agencies namely, construction of facilities, recruitment of health workers, and provision of drugs and other supplies. However, respondents indicated that several facilities were not established according to the standards of Ministry of Health and district to enable safe and quality service provision. In certain settlements, facilities were almost unusable – dilapidated at the time they were handed over to the DHT’s. Some of these facilities could not be maintained due to lack of funds in the district budgets. Only in Arua and Moyo districts did respondents report that facilities were decent enough to support service delivery. However, even then several required extensive rehabilitation.

*“The aid agencies did many things including establishing health facilities and supplying medicines and supplies. This provided us with an opportunity to extend much needed services to underserved communities. However, many of the facilities were sub-standard. Several were either in semi-permanent structures or poor structures. We faced challenges on what to do with the facilities because they could not be refurbished due to inadequate funding for the district”. (Chief Administrative Officer)*

Respondents indicated that collaboration between the district authorities and aid agencies was a challenge throughout the refugee emergency. This affected health service delivery in and around the settlements after the repatriation exercise. During all phases of the emergency, the district authorities engaged aid agencies to discuss strategies to improve service delivery through better coordination and harmonisation of plans and health programs. However, these efforts had few results in all the districts. The DLGs and DHTs sought the assistance of the MoH to support service delivery. There were suggestions that aid agencies should have made funds and other forms of support available beyond the emergency phase.

There were opinions among respondents that in the lead up to the repatriation of refugees, the disengagement with aid agencies was not formally organised in all the three districts. The events leading up to the end of refugee assistance was not well planned and coordinated. This led to difficulties in transitioning of responsibilities and management of health services to the local authorities and health departments. Lack of formal and systematic handover of service delivery points and other resources meant that the districts were not adequately prepared to assume responsibility for health service delivery. This was attributed to inadequate capacity of the district authorities worsened by poor cooperation with aid agencies.

*“When it was time for refugee repatriation, our interactions and working relation with the NGOs, was challenging. Remember that the capacity of the district local government has always been weak. When the agencies were departing, there was very little opportunity for the districts and agencies to manage an orderly separation that could ensure the district health services took over formally and minimized service disruptions.” (Community Development Officer)*

### Organisational interdependency and interconnectedness

Respondents at managerial level in all the three districts mentioned that while the MoH provided oversight over health services, it provided minimal direction and support to the DLGs and DHTs regarding health services during and after the repatriation exercise in all the three districts. The DLGs and the DHOs developed plans, and made health service delivery decisions with minimal input from the MoH. The DLGs made consultations and informed the MoH about what was happening regarding health services delivery for refugees and host communities. The MoH generally provided policy direction and was mainly active in planning and developing of health policies to guide the provision of health services.

*“One challenge that we have had in the district is that there has was little direction and assistance from the Ministry of Health on the management of health services during the refugee emergency. Decisions regarding establishment of facilities, staffing and stocking facilities with drugs and other supplies have been made by the District Local Government and DHOs Office. While we followed national policies, certain issues required direction and action by the Ministry.” (District Health Officer)*

The respondents highlighted how the DLGs and DHTs in the region engaged each other to learn and share practical experiences regarding service delivery for refugees and host populations. Both administrators and technical staff from the three districts organised and held frequent meetings. Through these meetings and exchanges, the districts learnt how the others were approaching various aspects and challenges of health service delivery. The meetings and linkages established benchmarks for engaging aid agencies and the government on how to improve provision of services.

*“As the district administration we organized and held periodical meetings to discuss approaches, opportunities and challenges in responding to the refugee crises including health services. We learnt from each other, various experiences, and ways of managing health service delivery for refugees and host communities. The technical departments were very more prominent in holding the meetings. Through these discussions we were able to learn and decide how to work with NGOs and the government to support refugees and host communities” (Chief Administer Officer)*

## Funding

The main source of funding for health services delivery in the three districts was through the Primary Health Care (PHC) grants disbursed by the central government to facilitate the implementation of the Basic Package of Essential Health Services. Respondents indicated that the PHC grants was provided to DLGs and remitted to health facilities to facilitate services delivery. The PHC funds are provided with emphasis on ensuring access and quality of services for both populations. It was observed that the PHC grants have been used to support all facilities including public and private not for profit health facilities including those established to provide services to refugees. The grants covered wage and non-wage expenses, transitional development such as outreach and public health community sensitization campaigns. Additionally, the funds were used for limited but specific support for the rehabilitation of health facilities.

The PHC grant supported service delivery in all facilities including those established in refugee settlements. To receive the PHC grant, the health facilities had to be accredited by the MoH based on evaluations of the DLG. All the health facilities in Arua and Moyo districts received the PHC grant and other forms of support from the central government. However, in Adjumani two health facilities were not accredited and did not receive any form of support including the PHC grant. The DLGs and the DHTs made arrangements to ensure that the two non-accredited health facilities were supported from other funding sources that were available to all the other health facilities.

While general support for the refugee emergency declined or ceased in the three districts following repatriation, Adjumani district continued to receive limited specific support. The district continued to host refugees in two settlements. The UNHCR supported the district by recruiting and providing salaries for four health workers; two midwives and a nurse at the general hospital and the two health facilities that continued to provide health services to refugees. Additional support provided included the purchase and maintenance of an ambulance to support referral services from the facilities serving refugees to the district hospital. However, several other non-governmental organizations provided support to health facilities that continued to serve refugees. Aid agencies ceased supporting and funding refugee assistance services in all other health facilities in the district.

The three districts received funding, technical and other forms of support from other agencies. While funding and other forms of assistance provided was not related to the refugee emergency, it supported service delivery in all health facilities including those that continued to serve refugees. Support provided include training and strengthening health management information systems, HIV/AIDS service delivery, child health and immunization programmes, water and sanitation and nutrition programmes.

*“Official support from aid agencies ended with the repatriation of refugees in this district. But we have NGOs that came in to support the district with funding and other forms of technical assistance. These were not related to protection and assistance to refugees, but they also supported health centres that continued to serve refugees as well.” (District health officer)*

While external funding and other forms of support ceased in Arua and Moyo districts, health service delivery did not stop in all facilities supported by aid agencies. This, despite challenges of inadequate medicines and other supplies, and the shortage of health workers in the three districts. This was pointed out that while government funding was inadequate, it minimized interruptions in service delivery and facilities remained operational.

*“Health service delivery was affected as funding and support by aid agencies reduced. This was more difficult with low funding from the central government. As the district health authority, we have a mandate to the communities we serve regardless of the conditions. Support by NGOs was important, but this did not mean we stop meeting the health needs of our people. We made several efforts with routine funding to ensure services delivery was not affected.” (District Health Officer)*

### Health services

Health services were provided in most refugee established facilities in all the three districts. Services were provided to both refugees and host populations following the repatriation exercise and departure of aid agencies. Health facilities provided basic preventive, promotive, and curative services. Health services were provided free of charge to all populations in all the facilities in the three districts. However, coverage of health services reduced in several formerly refugee established facilities. This, respondents attributed to the reduction in drugs and supplies and lack of health workers. Only one refugee established facility in Adjumani district continued to serve refugees and maintained high coverage levels.

*“While services delivery continued in all the facilities, service coverage was not the same as during the peak of refugee emergency. Many of the refugee facilities in the district struggled to serve the catchment populations due to reduced support by aid agencies. It is only one facility that continued to serve sizable numbers of refugees that maintained high coverage levels.” (District Planner)*

The DHT in all the three districts restructured health facilities in the former refugee settings (Table 2). In Moyo and Arua districts, health facilities established for refugees were not closed. While in Adjumani district, four facilities were restructured. Two facilities were closed while two were transferred to new locations. The two health facilities were closed due to two reasons. First, the demographic change meant that the catchment population of the health facility had drastically reduced due to the departure of refugees. Secondly, the poor condition of the health facilities as the structures were largely semi-permanent and in near dilapidated state. Other reasons identified include inadequate capacity including lack of health workers and shortage of medicines due to reduced support by aid agencies.

**Table 2:** Distribution of health facilities in Adjumani, Arua and Moyo districts following the repatriation of refugees

District	Total number of health facilities	Total number of host health facilities	Number of health facilities for refugees	Number of health facilities closed
Adjumani	33	13	17	4
Arua	71	63	8	0
Moyo	41	37	4	0
Total	145	116	29	4

^*^Source: Data retrieved from Annual District Health Office reports in Adjumani, Arua and Moyo districts, 2010–2015

Health services delivery was based on the integrated approach in facilities where refugees remained after the repatriation exercise. Both refugees and hosts received health services from the same facilities and providers. In Adjumani district, health services for all populations were provided through public health facilities. These health facilities continued to be supported by the aid agencies to augment government funding. The integration of health services minimized dual service delivery structures. Integration ensured that all efforts and interventions provided by all partners were channelled through the District Health Services.

*“At the end of the crises, all health services for refugees and hosts populations were combined. The integration strategy was a very important component of hosting refugees in the districts. This ensured that all efforts by all health partners were directed to the already established structures and facilities. This made the DHO withstand all the effects of emergency health assistance on healthcare in the district.” (District Planner)*

## Community and socio ecological conditions

### Community embeddedness

The respondents described how host communities organised themselves to address the challenges affecting health services in their community’s following the repatriation exercise. Community members had witnessed improvements in health services during the presence of refugees through the support of aid agencies. This encouraged host community members who organized themselves, pooled funds and purchased an ambulance to serve one health facility in Moyo district. The ambulance served the facility referral services transferring patients to hospitals in the sub-county. The acquisition of the community ambulance strengthened health services in the sub-county and surrounding areas.

*“As a result of hosting refugees and support of NGOs, the community mobilized themselves to solve their health problems. In fact, one community mobilized themselves, collected money and bought an ambulance to serve their health facility for referrals. This eventually helped the whole sub-county. This community believed that they should be able to withstand challenges and provide opportunities to improve their lives.” (Refugee Health Focal person)*

Community members were involved in decision making regarding service delivery during all stages of the refugee emergency. Specifically, respondents mentioned that the involvement of members of the health facility management committees in decisions regarding health services was vital in leveraging community buy in decisions and changes that the DLG made.

*“During the process of establishing health services for refugees including constructing this facility, we involved community members in discussions, regarding service delivery. This provided an opportunity to establish ownership of members especially regarding decisions on location and management of facilities since some were not yet operational by the government.” (Refugee Health Focal Person)*

### Policy environment

Health services were delivered based on several national policy frameworks in the country. The Self Reliance Strategy (SRS) was the key  document guiding specific issues relating to health service delivery for refugee and host community. The SRS aimed to address the challenges of protracted displacement of refugees in Uganda. The districts relied of the SRS to inform response to and support refugees. Three pillars of the strategy pursued by the districts including equity, dialogue, and mutual support that led to community self-reliance and inclusion of refugees in local government systems, such as for public health and nutrition services provision. Additionally, the SRS strategy promoted the integration of health and other social services for refugees and host communities.

## Discussions

This study explored the sustainability of humanitarian health assistance in the west Nile refugee hosting districts in Uganda. The study revealed that following the repatriation, the district health officer assumed overall responsibility for health services delivery for both refugees and host communities in all the three districts respectively. To minimize disruptions, the DHT in all the three districts reorganized health services in all former refugee health facilities. These included restructuring health facilities in former refugee settlements. Health workers were recruited and re-trained to soar up understaffed health facilities. Health services for refugees and host populations were delivered through public health facilities by the same providers. In all the three districts, funding for health services was through the PHC grant, with less or no support from aid agencies.

Following repatriation of refugee and departure of aid agencies, funding for health services in all the districts, was mainly provided through PHC grants by the central government. The PHC funds were used to provide basic health services including, preventive, and curative. Limited funding was provided by the aid agencies particularly for districts that continued to host refugees. The PHC is a considered a sustainable for of health financing. Sustainable financing is essential to ensure effective and efficient health systems. This is critical in emergency settings where inadequate funding and short-term nature of assistance mechanisms affect health service delivery. Government revenue, mostly generated through taxes are the main source of funding for health systems in many countries. However, funding of health services from taxes and other domestic revenue remains low in many low income countries (McIntyre, Obse et al. 2018). Despite this challenge, government revenue may offer sustainable means of funding health services in refugee settings. This is evidenced by policy developments in Uganda focusing on developing integrated services. These include the Health Sector Integrated Response Plan (HSIRP) [16] that provides strategic focus for the sector and how it contributes to the National Development Plan (NDP) 2015/16–2019/2020 NDP II [19]. These efforts fall under broad international and national goals of strengthening and ensuring sustainability of health programs in refugee settings.

The study shows that during the early phase of the refugee assistance, the DLGs and aid agencies made efforts to ensure all interventions were planned and implemented with long-term focus. This was demonstrated by the establishment of health facilities where permanent structures were built in several areas in the three districts. While this was not uniform practice, the findings show a focus on long-term sustainability by the DLG and aid agencies. In Adjumani district, however, our findings show that more health facilities were established for refugees than the government health facilities for the refugees population during the emergency. These health facilities however served both populations. As suggested by [28], given the protracted nature of refugee emergencies, there is need for long-term investment in sustainable systems and infrastructure including health care services. This is critical for countries such as Uganda that has been hosting refugees for several decades. Incorporating sustainability to assistance programmes ensures that when refugees arrive in the country and the host governments provide protection and assistance, the interventions not only serve in the emergency period but also in the long-term. This not only enhances sustainability of interventions but improves overall outlook and future support to refugees by governments in host countries.

The history of refugee crises in Uganda and responses by the government, aid agencies and the international community to protracted displacement, demonstrate that these are not isolated, short-term events that can be addressed using quick interventions followed by easy exit strategies. In this study, aid agencies have been operating and providing protection and assistance including water, basic health services and temporary shelter for decades. While the evidence of the effectiveness of these protection and assistance programmes is limited, most approaches used currently do not provide an adequate solution for addressing the medium and long-term needs and priorities of displaced populations and host communities. It is therefore critical to address questions on how to best provide more developmental focused programmes and services while preparing for a gradual sustainability.

The pursuit for sustainability of humanitarian assistance programmes presents policy makers and practitioners with challenges. If governments, aid agencies, and development partners adopt approaches that aims to ensure the sustainability of assistance programs, several considerations should be made. It is essential to develop plans that facilitate the pursuit of sustainability of humanitarian assistance programmes. This plan should include an assessment of needs of refugees and host population, the weaknesses, strengths and opportunities of current structures and service delivery systems before the initiation of any intervention. These should be followed by continuous measurement of accomplishments made during and at the end of the crises. While there is limited evidence to suggest that aid agencies pursued sustainability, several efforts and interventions made by the DLGs during the design, planning and implementation of refugee’s assistance programmes ensured interventions endured in the west Nile districts.

In this study, health services delivery for refugees in all the three districts except for Arua, were based on vertical assistance programmes for several years. Based on their primary mandate, aid agencies planned and mobilized resources to support health services for refugees in Adjumani and Moyo districts. While the departure of aid the agencies at the end of emergency did not lead to serious disruptions in health service delivery, several structures and services did not operate normally. For example, in Adjumani district, health services were re-organized with health facilities being relocated or closed following the repatriation of refugees. This was because of poor structure and reduction in the catchment population of the facilities. This finding emphasizes the need for developing exit strategies that ensure disruptions to services are minimized [30]. Findings from previous studies show that compared to integrated programmes, vertical programmes are less likely to be sustained (Haidari, Zaidi et al. 2014). Vertical programmes are value driven offering limited opportunities for sustainability and often have negative spill over effects on health systems and non-targeted populations [1]. Many vertical interventions place emphasis on resources and activities with clear goals and are therefore less likely to attract indigenous funding exposing interventions to failures when external funding ceases [3].

For decades, the question of sustainability has become central in debates around aid effectiveness and efficiency of humanitarian assistance programmes [4]. Several Frameworks, and assessments tools have been developed for analysing the sustainability of programmes [8]. However, there is limited evidence regarding efforts by governments and the humanitarian system to strengthen the sustainability of interventions during planning, designing and implementation of humanitarian assistance programmes. Besides for decades, the broader humanitarian system has placed little emphasis on addressing sustainability issues during planning and implementation of interventions. Further, in cases where country and sub-national systems have little input in the design, planning and implementation of programmes, the chances of sustainability are minimal. To ensure the sustainability of interventions, it is important to integrate assistance programmes into local systems.

This study has some limitations. First, the study is limited to the perspectives of respondent at the district level including local government personnel and NGO professionals as our respondents. While we tried to ensure a broad range of perspectives to inform the study, we could not reach other important stakeholders at national level. The study participants interviewed served as ‘proxy informants’, for the categories of location, professions, types of services. In addition, data were collected for a short time. We therefore may have missed important longitudinal perspectives that are critical when assessing sustainability. We therefore recommend further studies with longer follow-up in the field, including national level stakeholders, local partners, and community members.

## Conclusions

The findings of this study add to the limited research on the sustainability of health services in humanitarian settings. This study showed that health authorities in the three districts continued to provide health services to both refugees and host populations beyond the refugee emergency. Our study showed that while humanitarian health services are not designed for long term sustainability, several interventions have endured. Overall, the embeddedness of the refugee health services in the district health systems ensured health services were sustained. It is important to strengthen the capacity of the local service delivery structures and ensure that refugee health services are integrated as a key strategy of promoting sustainability.

## Data Availability

The data used in this study is available upon request to the authors. However, the request should be within the research and ethics restrictions of the Higher Degrees and Research Ethics Committee of Makerere University School of Public Health and Uganda National Council for Science and Technology.

## Abbreviations

CSSA: Child Survival sustainability assessment
DHO: District health office
DHS: District health services
DLG: District local government
EMA: Emergency medical assistance
IHA: International humanitarian assistance
MoH: Ministry of health
NGO: Non-governmental organisation
ODA: Overseas development assistance
PHC: Primary health care
SDG: Sustainable development goals
UN: United Nations
UNHCR: United Nations high commission for refugees
